# Genome-Wide Identification and Expression Analysis of the *SRS* Gene Family in *Hylocereus undatus*

**DOI:** 10.3390/plants14203139

**Published:** 2025-10-11

**Authors:** Fanjin Peng, Lirong Zhou, Shuzhang Liu, Renzhi Huang, Guangzhao Xu, Zhuanying Yang

**Affiliations:** College of Coastal Agricultural Sciences, Guangdong Ocean University, Zhanjiang 524088, China; pengpengpengpao@stu.gdou.edu.cn (F.P.); 18894837003@stu.gdou.edu.cn (L.Z.); liushuzhangcofco@163.com (S.L.); 13356516730@stu.gdou.edu.cn (R.H.)

**Keywords:** pitaya, the *SRS* gene family, whole-genome identification, callus development, gene expression analysis

## Abstract

SHORT INTERNODE (SHI)-Related Sequence (SRS) transcription factors play crucial roles in plant growth, development, and stress responses and have been extensively studied in various plant species. However, the molecular functions and regulatory mechanisms of *SRS* genes in the economically important tropical fruit crop pitaya (*Hylocereus undatus*) remain poorly understood. This study identified 9 *HuSRS* genes in pitaya via bioinformatics analysis, with subcellular localization predicting nuclear distributions for all. Gene structure analysis showed 1–4 exons, and conserved motifs (RING-type zinc finger and IXGH domains) were shared across subclasses. Phylogenetic analysis classified the *HuSRS* genes into three subfamilies. Subfamily I (*HuSRS1*–*HuSRS4*) is closely related to poplar and tomato homologs and subfamily III (*HuSRS6*–*HuSRS8*) contains a recently duplicated paralogous pair (*HuSRS7*/*HuSRS8*) and shows affinity to rice SRS genes. Protein structure prediction revealed dominance of random coils, α-helices, and extended strands, with spatial similarity correlating to subfamily classification. Interaction networks showed HuSRS1, HuSRS2, HuSRS7 and HuSRS8 interact with functional proteins in transcription and hormone signaling. Promoter analysis identified abundant light/hormone/stress-responsive elements, with *HuSRS5* harboring the most motifs. Transcriptome and qPCR analyses revealed spatiotemporal expression patterns: HuSRS4, HuSRS5, and HuSRS7 exhibited significantly higher expression levels in callus (WG), which may be associated with dedifferentiation capacity. In seedlings, *HuSRS9* exhibited extremely high transcriptional accumulation in stem segments, while *HuSRS1*, *HuSRS5*, *HuSRS7* and *HuSRS8* were highly active in cotyledons. This study systematically analyzed the characteristics of the *SRS* gene family in pitaya, revealing its evolutionary conservation and spatio-temporal expression differences. The research results have laid a foundation for in-depth exploration of the function of the *SRS* gene in the tissue culture and molecular breeding of pitaya.

## 1. Background

Pitaya (*Hylocereus undatus*), native to Central and Northern South America, has been widely cultivated in Southeast Asia, Australia, and China. Valued for its rapid growth rate and strong resistance to environmental stresses, this fruit crop nonetheless faces challenges in genetic improvement due to natural hybridization and complex genetics, leading to slow breeding progress. To address this, genomic studies are essential to map the pitaya genome and identify key functional genes—including those underlying its desirable traits of fast growth and robust stress resistance. This will enable marker-assisted selection, improving breeding efficiency and accelerating the development of superior cultivars that retain or enhance these advantageous characteristics. This approach holds significant scientific and commercial value for pitaya improvement [1,2].

The SRS family is a plant-specific group of transcription factors that play diverse regulatory roles in plant growth, development, and responses to adverse environmental stresses. These factors contribute to the regulation of plant growth and developmental processes through their involvement in the biosynthesis of plant hormones and the modulation of signal transduction pathways [3,4,5,6]. Among model plants, thale cress (*Arabidopsis thaliana)* has been most extensively studied for *SRS* gene functions. Notably, the *SHI* gene negatively regulates gibberellin (GA) signaling, thereby influencing inflorescence stem elongation and flowering time [7]. Similarly, STY1 and other SHI/STY family members function as transcriptional activators that regulate auxin homeostasis, particularly during apical meristem development. These findings collectively demonstrate the crucial involvement of *SRS* genes in coordinating phytohormone crosstalk to orchestrate plant growth and developmental processes [8]. *LRP1* regulates the lateral root development of *Arabidopsis* by modulating auxin signal transduction [9,10]. Overexpression of *AtSRS7* has been shown to disrupt jasmonic acid (JA) signaling, resulting in multiple developmental abnormalities including plant dwarfism, leaf curling, delayed anther dehiscence, and impaired floral organogenesis [11]. These phenotypic manifestations, coupled with the established roles of other *SRS* family members in gibberellin and auxin regulation, collectively demonstrate that the *SRS* gene family serves as a critical modulator of both developmental processes and hormonal responses in *Arabidopsis thaliana*.

Emerging studies have revealed crucial regulatory roles of the SRS gene family in monocotyledonous plants. In rice (*Oryza sativa*), OsSHI1 modulates tiller number and grain size by binding to the T/GCTC-TAC *cis*-element in promoters of key developmental regulators (*OsTB1* and *OsDEP1*), thereby suppressing *IPA1* expression [12]. Similarly, maize (*Zea mays*) LRP1, another SRS family member, governs lateral root morphogenesis [13]. The *SRS* gene family exhibits critical regulatory roles across diverse dicot species, participating in multiple developmental and stress response pathways. In tomato (*Solanum lycopersicum*), genome-wide characterization has identified eight *SlSRS* members that significantly influence floral organogenesis and phytohormone signaling [14]. Similarly, in soybean (*Glycine max*), GmSRS18 mediates drought and salt stress adaptation through modulation of stress-responsive pathways [15]. Alfalfa (*Medicago sativa*) SRS members demonstrate essential functions in growth regulation, while in cotton (*Gossypium hirsutum*), the salt-inducible GhSRS21 acts as a negative regulator of salinity stress responses [16]. These findings collectively highlight the functional versatility of *SRS* genes in coordinating developmental programs and environmental adaptation in dicot crops [17].

Despite the functional studies of the *SRS* gene family having made certain progress in various plants, the identification and analysis of the *SRS* gene family in pitaya have not been reported yet. This study aims to identify the members of the *HuSRS* gene family, and analyze their physicochemical properties, gene structures, phylogenetic relationships, as well as expression patterns in different tissues, so as to reveal their functions in the growth and development of pitaya.

## 2. Methods

### 2.1. Identification and Physicochemical Properties of HuSRS Gene Family Members

To identify candidate *SRS* family genes, we constructed a local BLAST database (TBtools-IIv2.357) using the gene sequences of the *Arabidopsis* and rice *SRS* families as seed sequences, which was subsequently aligned against the publicly available Pitaya Genomic Database (Chen et al., 2021) [18]. The hidden Markov model (PF05142) encoding the SRS protein domain was obtained from the Pfam database. The domains of candidate *SRS* genes were analyzed through three major databases: SMART (http://smart.embl.de/ (accessed on 12 March 2025)) [19], CDD-search (https://www.ncbi.nlm.nih.gov/cdd/ (accessed on 12 March 2025)) [20], and PFAM (http://pfam.xfam.org/ (accessed on 12 March 2025)). Eventually, *SRS* family genes in pitaya were obtained and named *HuSRS1* to *HuSRS9* based on their chromosomal locations.

The physicochemical properties of HuSRS proteins, including the number of amino acids, isoelectric point, and aliphatic index, were predicted and analyzed using the ProtParam tool from the ExPASy online database (http://www.expasy.org/ (accessed on 14 March 2025)). Additionally, the subcellular localization of these proteins was predicted via the Cell-PLoc 2.0 online software (http://www.csbio.sjtu.edu.cn/bioinf/Cell-PLoc-2/ (accessed on 14 March 2025)) [21].

### 2.2. Chromosomal Localization and Collinearity Analysis of the HuSRS Gene Family

**Figure 1:**
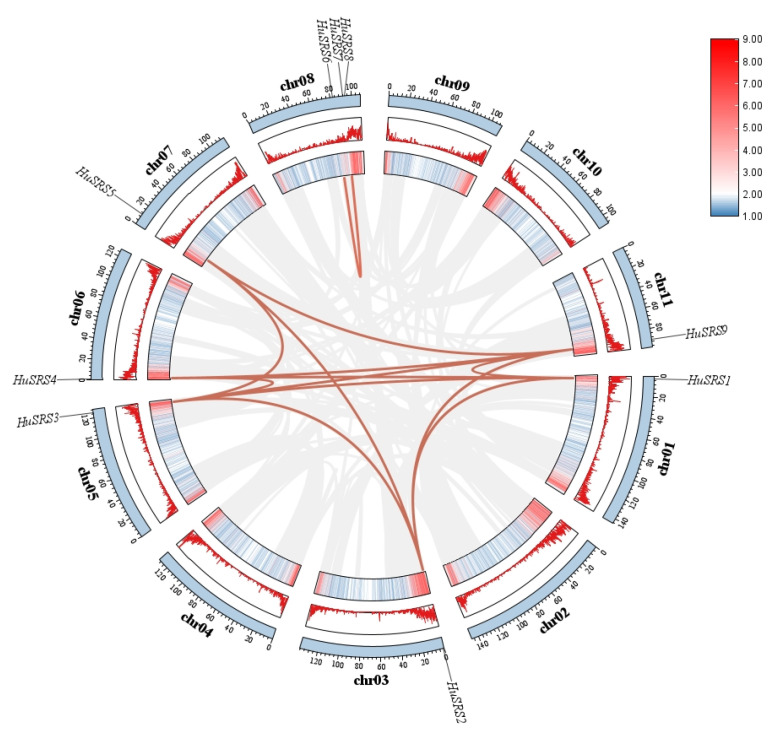
Chromosomal localization and duplication analysis of *HuSRS* genes. Genome-wide collinearity analysis was performed using MCSCANX, and the Circos plot was generated with TBtools-IIv2.357. The outer ring depicts the 11 chromosomes represented as blue arcs, with gene IDs labeled above each chromosome. The middle and inner rings show gene density in heatmap and linear plot formats, respectively. Gray lines in the background indicate syntenic blocks across the pitaya genome, while red lines highlight duplicated *HuSRS* genes. The gradient from blue to red in the data bars represents increasing gene density.

The chromosomal physical positions of *HuSRS* genes were determined by parsing the *Hylocereus* genome GFF annotation data using TBtools-IIv2.357 software. Duplicate gene pairs within the *HuSRS* family were identified using MCScanX v1.0.0 software [22], and their chromosomal distribution patterns were visualized via the Advanced Circos function in TBtools-IIv2.357 [23]. Additionally, the Ka/Ks Calculator plugin in TBtools was employed to compute the nonsynonymous (Ka) and synonymous (Ks) substitution rates for the identified gene pairs. (See Figure 1).

### 2.3. Conserved Motifs and Gene Structures of the HuSRS Gene Family

Conserved motifs within the HuSRS proteins were identified using MEME Suite v4.12.0 [24] with the following parameters: maximum number of motifs set to 10, motif width constrained between 6 and 50 amino acids. TBtools-IIv2.357 software was employed to visualize these conserved domains. Additionally, gene structure diagrams depicting intron-exon organization were generated using TBtools based on the *Hylocereus* genome annotation data.

### 2.4. Phylogenetic Tree Construction of the HuSRS Gene Family

A phylogenetic tree was reconstructed using SRS protein sequences from six species: pitaya (*Hylocereus undatus*), tomato (*Solanum lycopersicum*), poplar (*Populus trichocarpa*), rice (*Oryza sativa*), maize (*Zea mays*), and *Arabidopsis thaliana* [25]. The analysis was performed in MEGA 11 [26] using the Neighbor-Joining (NJ) method with 1000 bootstrap replicates. The resulting tree was visualized and annotated using the online tool iTOL (https://itol.embl.de/ (accessed on 17 March 2025)) [27]. The SRSs from the six species are detailed in Supplementary Table S1.

### 2.5. Secondary and Tertiary Structural Prediction and Protein–Protein Interaction Analysis of the HuSRS Gene Family

Secondary structure prediction of HuSRS-encoded proteins was performed using the PRABI online tool (https://doua.prabi.fr/software/cap3 (accessed on 17 March 2025)), with results systematically compiled and summarized. Tertiary structure modeling was conducted via SWISS-MODEL (https://swissmodel.expasy.org/interactive (accessed on 17 March 2025)) [28], while protein–protein interaction (PPI) networks were analyzed using the STRING database (https://string-db.org/ (accessed on 17 March 2025)) [29].

### 2.6. Temporal and Spatial Expression Patterns and Cis-Regulatory Element Analysis of the HuSRS Gene Family

Transcriptome data were obtained from the publicly accessible Pitaya Genomic Database to analyze the spatio-temporal expression patterns of *HuSRS* genes across different floral and fruit-related tissues (flowers, flesh, and peel) of *Hylocereus undatus* ‘Jingdu No.1’. Expression heatmaps were generated using TBtools. Promoter sequences (2000 bp upstream of the translation start site) of *HuSRS* family genes were extracted, and *cis*-acting elements were predicted using the Search for CARE tool in the PlantCARE [30] database with a default threshold (E-value < 1 × 10^−5^). Visualization heatmaps of *cis*-elements were constructed via TBtools.

### 2.7. Physiological Index Determination of Pitaya Callus and Spatial Expression Quantitative Analysis of HuSRS Gene Family

#### Plant Materials and Treatments

This study used seeds of *Hylocereus undatus* ‘Jingdu No.1’ as the experimental material. The seeds were collected from fruits of different mother plants of the same commercial variety, pooled, and then randomly selected for experimentation to account for potential individual variation while maintaining genetic background consistency. Seeds were sterilized using a three-step disinfection protocol: immersion in 75% ethanol for 30 s, followed by treatment with 2% sodium hypochlorite for 2.5 min, and then 0.1% mercuric chloride for 10 min. After five rinses with sterile water, the surface-dried seeds were inoculated onto a seed germination solid medium (1/2 MS + 30 g/L maltose + 20 g/L sucrose + 0.3 g/L activated charcoal + 0.75% agar) for aseptic germination to obtain seedlings. Uniform six-week-old seedlings derived from the germinated seeds were selected, and their stem segments, cotyledons, and root tissues were collected and stored at −80 °C for subsequent experiments. Among these, stem segments (approximately 3–4 cm in length and 0.3–0.5 cm in diameter) from the six-week-old uniform seedlings were used for callus induction. The stem segments were cut into 0.5 cm sections and placed horizontally on callus induction medium (1/2 MS + 0.6 mg/L TDZ + 0.8 mg/L KT + 0.5 mg/L 2,4-D + 10 g/L maltose + 20 g/L sucrose + 0.75% agar). All cultures were maintained in darkness at 25 °C, and the medium was refreshed once after 3 weeks. After 6 weeks of culture, well-developed callus formed on some of the explant stem segments. The resulting tissue materials were classified into two types: WG: Well-Grown, Densely Proliferative Compact Clumps (After 6 weeks of culture, the callus formed dense-textured clumps with vigorous proliferation). CK: Control Group with Sparse Undifferentiated Clusters (After 6 weeks of culture, no callus was induced, or only sparse, loosely structured cell clusters formed). After sampling, the tissues were immediately stored at −80 °C for future use.

### 2.8. Coomassie Brilliant Blue G-250 Staining Method

The soluble protein content of the samples was quantified using the Coomassie Brilliant Blue G-250 method [31], with bovine serum albumin (BSA) as the standard. Absorbance was measured at 595 nm using a microplate reader. The resulting regression equation is y = 0.0063x − 0.0693 (R^2^ = 0.9979).

### 2.9. Determination of Relative Electrolyte Conductivity (REC)

Membrane integrity damage was assessed by measuring the relative electrolyte conductivity (REC). Callus tissues were incubated in distilled water on a shaker, and the conductivity was measured before and after boiling. Each treatment included three biological replicates. Calculate the relative electrolyte conductivity (REC) using the formula [32]:

REC= (S_1_ − S_0_)/(S_2_ − S_0_) × 100%.

### 2.10. Expression of the HuSRS Genes in Pitaya Callus and Seedlings

The expression patterns of the *HuSRS* genes in pitaya callus (CK, WG) and seedlings (stem segments, cotyledons, roots) were analyzed using quantitative real-time PCR (qRT-PCR). Primers for qRT-PCR were designed using the National Center for Biotechnology Information (NCBI, https://www.ncbi.nlm.nih.gov/ (accessed on 11 April 2025)) and synthesized by Sangon Biotech. *HuActin* was chosen as the reference gene due to its stable expression across tissues and treatments, as validated by transcriptome stability analysis [33]. Total RNA was extracted using the SteadyPure Plant RNA Extraction Kit (Accurate Biotechnology, Changsha, China) according to the manufacturer’s instructions. First-strand cDNA was synthesized from 1 μg of total RNA using the Hifair^®^ II 1st Strand cDNA Synthesis SuperMix for qPCR (Yeasen Biotechnology, Shanghai, China). The obtained cDNA was diluted 10-fold with nuclease-free water and used as the template for qPCR. Each 20 μL qPCR reaction contained 10 μL of Hieff^®^ qPCR SYBR Green Master Mix (No Rox) (Yeasen Biotechnology, Shanghai, China), 0.8 μL each of forward and reverse primers (10 μM), 2 μL of diluted cDNA template, and 6.4 μL of nuclease-free water. The amplification protocol was performed as follows: initial denaturation at 95 °C for 5 min; 40 cycles of denaturation at 95 °C for 5 s, and annealing/extension at 60 °C for 30 s; followed by melt curve analysis from 65 °C to 95 °C. Three biological replicates were included for each sample, and gene expression analysis was strictly carried out following the qRT-PCR procedures. The relative expression levels were calculated using the 2^(−ΔΔCt) method with *HuActin* as the reference gene. Statistical analysis was performed using GraphPad Prism 10.1.2. One-way ANOVA was applied to assess differences, with independent samples *t*-tests used to determine significance between CK and WG callus groups, and Dunnett’s multiple comparison test (*p* < 0.01) for differences among seedling tissues [34]. Primers used for RT-qPCR are listed in Supplementary Table S2.

## 3. Results

### 3.1. Identification of the HuSRS Gene Family Members and Analysis of Their Encoded Proteins’ Basic Physicochemical Properties

Through whole-genome scanning and bioinformatics analysis, nine members of the *HuSRS* gene family were identified and designated as *HuSRS1* to *HuSRS9*. The study revealed that the amino acid lengths of these genes ranged from 235 to 365, with molecular weights between 25.88 and 38.87 kD. Their isoelectric points (pI) varied from 6.92 to 9.13, indicating that HuSRS3 and HuSRS5 were acidic proteins, while the others were alkaline. The average hydrophilicity (GRAVY) values were all below 0, suggesting that all members are hydrophilic proteins.

Subcellular localization predictions showed that most of the nine family members were primarily localized in the nucleus. However, HuSRS3 and HuSRS4 were predicted to localize in the chloroplast, while HuSRS6 was predicted to be associated with the cell membrane (Table 1).

### 3.2. Chromosomal Distribution, Replication Events and Collinearity Analysis of the HuSRS Gene Family

Nine *HuSRS* genes were mapped to seven chromosomes of pitaya, with chromosome 8 harboring three genes. To explore the evolutionary history of *HuSRS* genes, collinearity analysis was performed. The results showed 13 pairs of homologous gene pairs (involving 8 *HuSRS* genes), all of which were products of segmental duplication events. This indicates that segmental duplication was the primary evolutionary force driving the expansion of the *HuSRS* gene family.

Using TBtools-IIv2.357 software to calculate the nonsynonymous mutation rate (Ka) and synonymous mutation rate (Ks) of each homologous gene pair, we found that the Ka/Ks ratios of *HuSRS* orthologous gene pairs were all less than 1. This result suggests that *HuSRS* genes have undergone purifying selection during evolution, exhibiting high conservation in sequence function(Figure 1).

### 3.3. Gene Structures and Conserved Motifs of the HuSRS Gene Family

**Figure 2:**
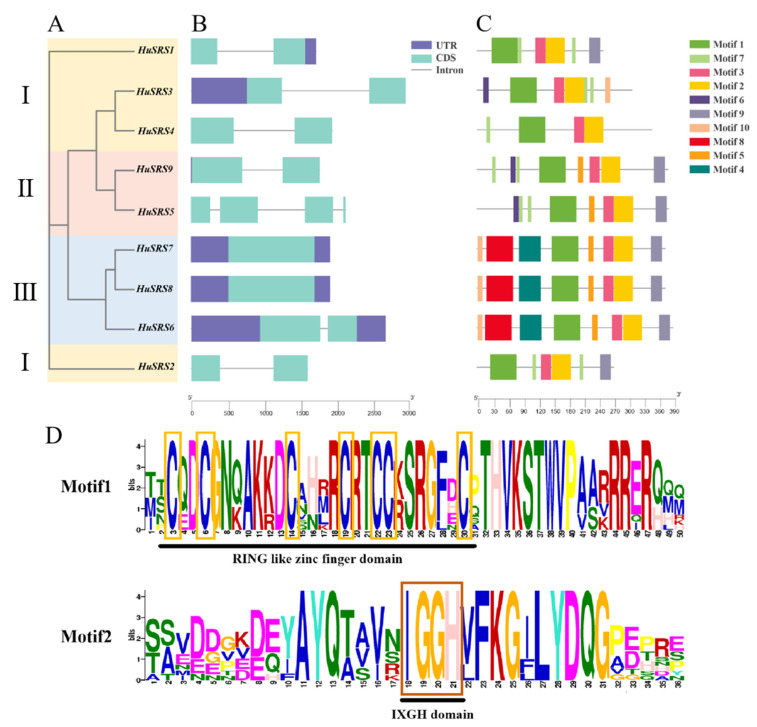
Gene structure and conserved motif distribution for *HuSRS* genes. (**A**) Neighbor-joining phylogenetic tree of *HuSRSs*; (**B**) *HuSRSs* gene structure; (**C**) *HuSRSs* conserved motif; (**D**) The most conserved Motif1, Motif2 in *HuSRSs*. TBtools was used to visualize the above results.

Based on the number and types of motifs, the phylogenetic tree was divided into three subclasses (Figure 2A). Analysis of the exon-intron structures of *HuSRS* genes (Figure 2B) showed that the number of exons in the *HuSRS* family ranged from 1 to 4, and both *HuSRS2*, *HuSRS4*, and *HuSRS5* lacked UTRs at both ends. Conserved motif analysis (Figure 2C) revealed that Class II members *HuSRS5* and *HuSRS9* contained seven identical conserved motifs, while Class III members *HuSRS6*, *HuSRS7*, and *HuSRS8* shared eight identical motifs. The number of conserved motifs in the nine *HuSRS* genes ranged from 4 to 8, with *HuSRS4* having the fewest (only four). Notably, Motif 1, Motif 2, and Motif 3 were common to the entire family. Most members within the same cluster exhibited similar motif compositions, suggesting functional similarities among genes in the same group. Furthermore, Motif 1 was identified to contain a RING-type zinc finger domain, which was present in all HuSRS proteins. Additionally, Motif 2 was found to possess an IXGH domain, also conserved across all HuSRS proteins (Figure 2D).

### 3.4. Phylogenetic Tree Analysis of the HuSRS Gene Family

**Figure 3:**
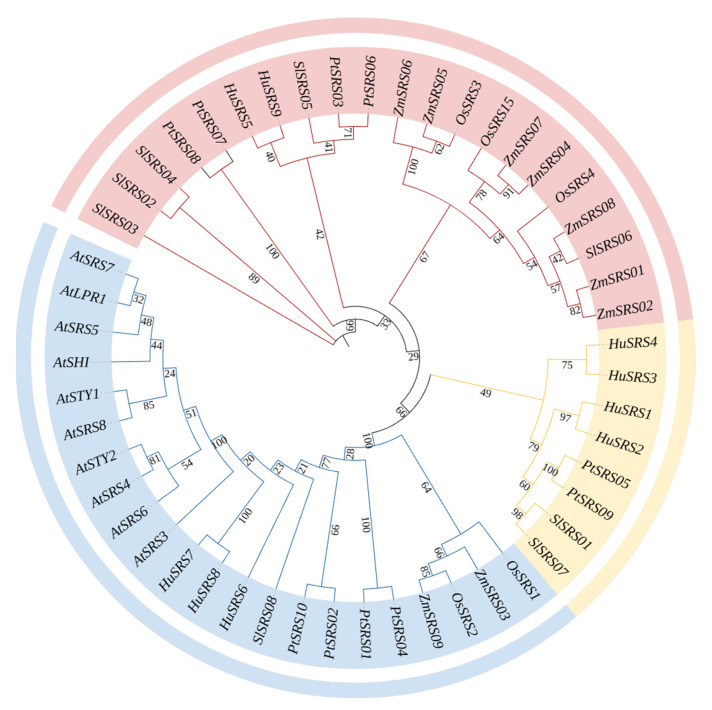
Phylogenetic relationships of *SRS* gene family among multiple species. A phylogenetic tree was constructed using the Neighbor-Joining (NJ) method based on SRS protein sequences from *Arabidopsis thaliana*, *Oryza sativa*, *Zea mays*, *Solanum lycopersicum*, *Populus trichocarpa*, and *Hylocereus undatus*, with 1000 bootstrap replicates. The tree was visualized and edited using MEGA11 software and further refined through the online platform iTOL. The different colored arcs represent distinct clades: Class I (yellow), Class II (pink), and Class III (blue), which is consistent with the coloring scheme in Figure 2.

A phylogenetic tree (Figure 3) was constructed using MEGA11 software by aligning amino acid sequences of *SRS* genes from *Arabidopsis thaliana*, *Oryza sativa*, *Zea mays*, *Solanum lycopersicum*, *Populus trichocarpa* and *Hylocereus undatus*. Based on the number and types of motifs, the phylogenetic tree was classified into three subclasses.

The evolutionary tree is clearly divided into two major branches: monocotyledons and dicotyledons. The *HuSRS* genes of pitaya are closely clustered with homologous genes from tomato and poplar, forming a highly supported dicotyledonous branch, which is consistent with its taxonomic classification. Notably, although *Arabidopsis SRS* genes belong to the dicotyledonous branch, they form an independent long branch, suggesting that they may have undergone accelerated evolution or functional divergence.

Based on phylogenetic relationships, the pitaya *SRS* genes can be divided into three subfamilies: The first subfamily includes *HuSRS1*, *HuSRS2*, *HuSRS3*, and *HuSRS4*. This subfamily is more closely related to homologous genes from poplar and tomato, indicating functional conservation among dicotyledonous plants. The second subfamily includes *HuSRS5* and *HuSRS9*, which may represent an ancient evolutionary lineage. These duplicated genes likely contributed to family expansion through functional divergence. The third subfamily includes *HuSRS6*, *HuSRS7*, and *HuSRS8*, with *HuSRS7* and *HuSRS8* exhibiting the highest support values, suggesting that they are a pair of paralogous genes produced by recent duplication events. The *SRS* genes in Arabidopsis have also expanded, but their evolutionary path is more independent, possibly due to family- or genus-specific adaptations.

The SRS genes of monocotyledons such as rice and maize form a separate cluster. The results indicate that pitaya SRS genes are evolutionarily conserved with homologous genes from other dicotyledons but significantly differ from those of Arabidopsis, reflecting divergent evolutionary pressures across lineages. Gene duplication events leading to subfamily differentiation have been a major driving force for the expansion of the pitaya SRS family, potentially providing an evolutionary basis for its unique traits.

### 3.5. Protein Structure and Protein–Protein Interaction Analysis of the HuSRS Protein Family

Secondary structure prediction of HuSRS proteins using the PRABI online tool revealed three structural components: α-helices (6.72–11.35%), extended strands (6.18–10.74%), and random coils (77.91–86.80%). The relatively stable content of these secondary structures suggests specific conserved characteristics (Table 2).

**Figure 4:**
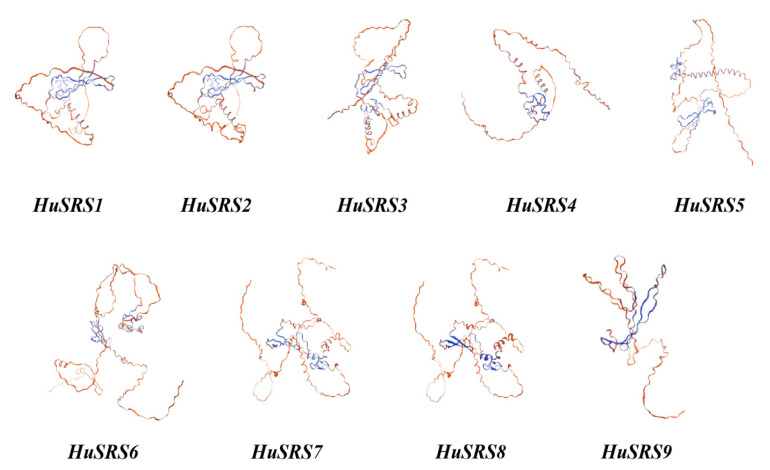
Prediction of HuSRS proteins tertiary structure. The structure was predicted using the SWISS-MODEL online platform, showing β-sheet, α-helix, and random coil structures.

Random coils constituted the major structural component of HuSRS proteins. Homology modeling via SWISS-MODEL (Figure 4) showed that HuSRS1 and HuSRS2 shared similar spatial structures, as did HuSRS7 and HuSRS8, while HuSRS4 and HuSRS5 exhibited shorter and simpler architectures. Proteins with similar spatial structures all belonged to the same subclass, indicating that sequence conservation determines structural similarity to some extent.

**Figure 5:**
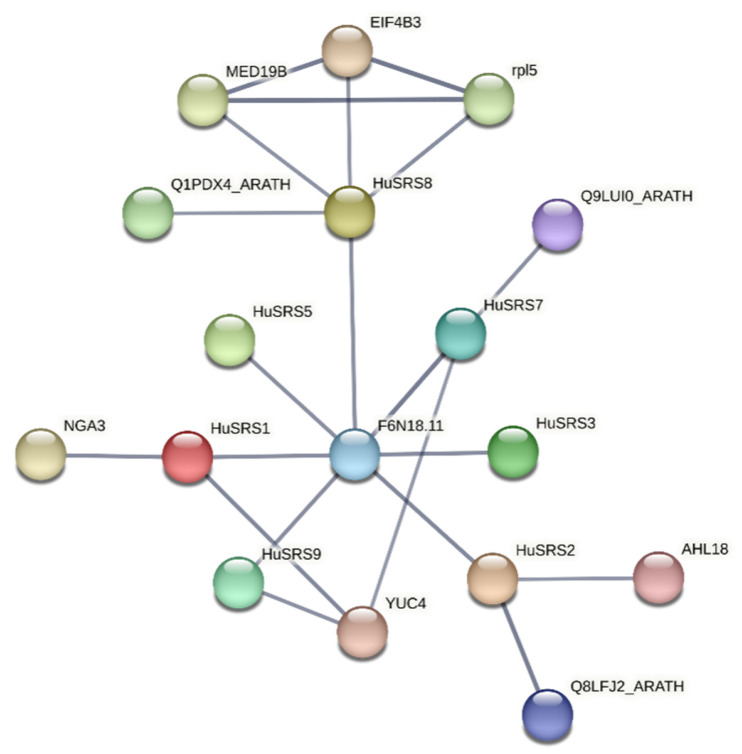
Protein–protein interaction analysis of HuSRS proteins.

To further explore the potential biological functions of pitaya HuSRS proteins, a protein–protein interaction (PPI) network was constructed based on Arabidopsis protein annotations. The prediction showed that HuSRS proteins formed a complex interaction network (Figure 5). F6N18.11 interacted with all family members, while YUC4—implicated in auxin biosynthesis, embryogenesis, seedling development, and essential for floral organ and vascular tissue formation—interacted with HuSRS1, HuSRS7, and HuSRS9. Notably, four key HuSRS proteins showed distinct functional associations: HuSRS8 is predicted to interact with MED19B, EIF4B3, rpl5, and Q1PDX4_ARATH; HuSRS2 associated with AHL18 and Q8LFJ2_ARATH; HuSRS7 interacted with Q9LUI0_ARATH; and HuSRS1 bound to NGA3.

### 3.6. Temporal-Spatial Expression Transcriptome and Cis-Regulatory Element Analysis of HuSRS Genes

**Figure 6:**
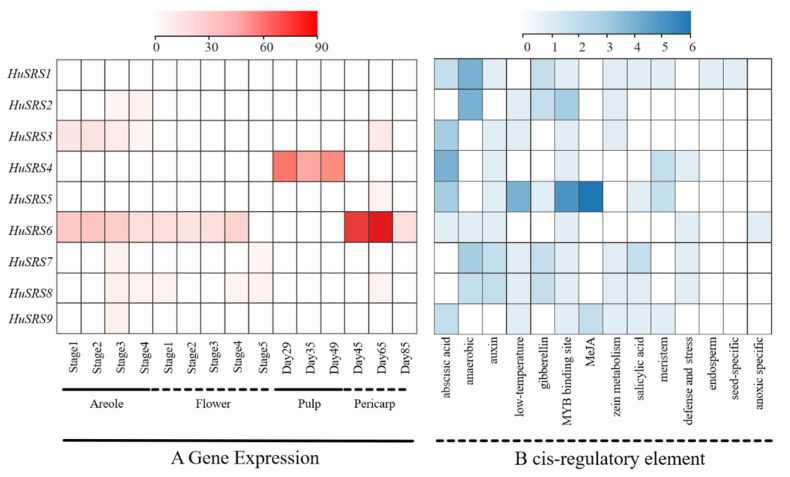
Spatio-temporal expression and cis-regulatory elements of *HuSRSs*. (**A**) Profiling of *HuSRSs* at various developmental stages of flowers and fruit tissues. (**B**) Abundance of cis-regulatory elements in promoters of *HuSRSs*. The red color scale represents log2(FPKM) expression values in the heatmap, while the blue color scale indicates the number of cis-regulatory elements.

Based on the published pitaya RNA-seq dataset, we conducted an in-depth analysis of *HuSRS* gene family expression profiles during the development of pitaya flesh, flowers, and peel across different stages. Except for *HuSRS5*, the other 8 genes were expressed during flower development, with *HuSRS6* showing a significant expression peak at the areole differentiation stage, followed by a gradual decline with flower maturation (Figure 6A). All expressed genes exhibited higher expression levels at the flower bud differentiation stage than at the blooming stage, suggesting their potential key regulatory roles in flower bud differentiation. During flesh development, only *HuSRS1*, *HuSRS4*, *HuSRS6*, and *HuSRS7* were expressed among the nine family members. Notably, *HuSRS4* showed a sharp expression increase at 29 days of flesh development, indicating its potential involvement in regulating critical processes at specific flesh development stages. In peel development, 8 genes except *HuSRS4* were expressed, with expression peaking at 65 days of development. Among them, *HuSRS6* maintained high expression throughout the peel development stage, suggesting its important function in regulating peel maturation.

Analysis of *cis*-elements in the *HuSRS* gene promoter region using PlantCare identified 361 *cis*-acting elements (excluding core promoter elements and those with unknown functions) (Figure 6B), mainly categorized into light-responsive, hormone-responsive, and stress-responsive elements, which were evenly distributed in each gene promoter. All genes contained light-responsive and hormone-responsive elements, indicating a close correlation between *HuSRS* gene family regulation and light/hormone signals. *HuSRS5* harbored the highest number of *cis*-acting elements (49). Light-responsive elements accounted for 69.2% (250), and hormone-responsive elements accounted for 12.7% (46), covering various hormone-responsive elements such as abscisic acid (ABRE), jasmonic acid (CGTCA-motif, TGACG-motif), etc., with *HuSRS5* containing 12 hormone-responsive elements, the highest among all. Additionally, the promoter sequences also contained growth and development-related elements such as meristem expression, anaerobic induction, and zein metabolism elements, showing differential distributions in different genes. For example, *HuSRS1* simultaneously contained endosperm and seed-specific regulatory elements, suggesting its potential role in seed development regulation. In summary, *HuSRS* genes participate in multi-level regulatory networks of light, hormones, stress, and growth development through the specific distribution of *cis*-elements.

### 3.7. Physiological Characteristics of Pitaya Callus and Quantitative Analysis of Temporal-Spatial Expression of HuSRS Gene Family

**Figure 7:**
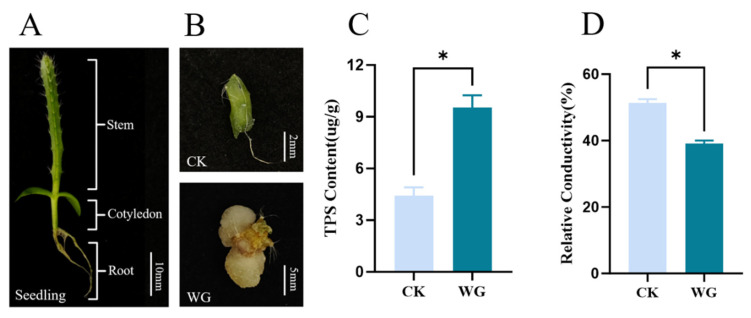
Analysis of physiological indices in dragon fruit callus. (**A**) Pitaya seedlings; (**B**) Pitaya callus; (**C**) Callus (CK, WG) Electrical Conductivity. (**D**) Total soluble protein content in callus (CK, WG). Asterisks indicate significant differences determined by independent samples *t*-test (*p* < 0.05).

Given that the transcriptome sequencing data only involved gene expression levels in pitaya flowers, flesh, and peel, this study used real-time fluorescent quantitative PCR (qPCR) to quantify the expression levels of *SRS* genes in cotyledons, stem segments, and root tissues of seedlings (Figure 7A) and in pitaya callus CK and WG (Figure 7B). Pitaya *ACTIN* gene was used as an internal reference gene to calculate relative expression levels, aiming to fill the gap in expression data of non-reproductive tissues and vegetative organs in the transcriptome.

Results of physiological indices showed that the average soluble total protein content was 4.42 μg/g in CK and 9.53 μg/g in WG (Figure 7C). The average conductivity was 51.6% in CK and 39% in WG. Compared with CK, WG showed characteristics of high protein content and low conductivity (Figure 7D). These physiological indices reflect the metabolic activity and membrane integrity of callus, which are indicative of its regeneration capacity.

**Figure 8:**
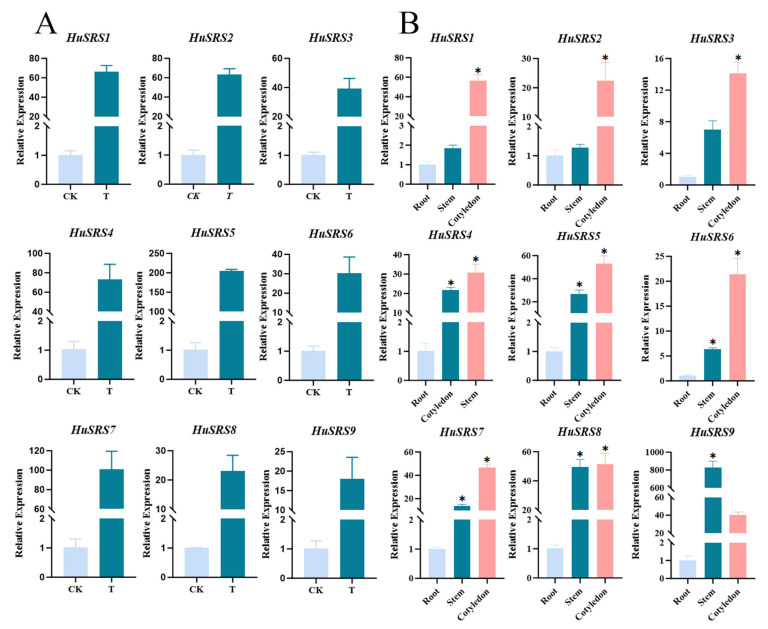
Analysis of spatial expression patterns of *HuSRS* genes. (**A**) Expression levels of *HuSRS* genes in CK and WG groups. (**B**) Expression levels of *HuSRS* genes in stem segments, cotyledons, and roots of dragon fruit seedlings. The expression level of *HuSRS* genes in roots was set as 1. Data represent the mean values of three independent biological replicates, with error bars indicating standard errors. * *p* < 0.05 indicates a statistically significant difference.

Significant differences were observed in the expression of *HuSRS* genes between CK and WG materials (Figure 8A). All *HuSRS* genes showed higher expression levels in WG than in CK. Notably, *HuSRS4*, *HuSRS5*, and *HuSRS7* exhibited high expression in WG, with relative expression levels exceeding 100. Combined analysis of *HuSRS* gene expression and physiological indices during plant tissue culture revealed a significant correlation between the physiological status and *SRS* gene expression levels in CK and WG. WG was characterized by high soluble protein content, low electrical conductivity, reflecting active cellular metabolism, stable membrane systems, and excellent water retention capacity. Callus in WG showed active proliferation but no obvious differentiation. The high expression of *SRS* genes under this state suggests their potential association with dedifferentiation processes. In contrast, the CK group displayed declined physiological functions, including weak metabolic activity, poor membrane stability, and an insufficient response to induction and differentiation signals.

The expression of *HuSRS* genes in vegetative organs (roots, stem segments, cotyledons) of pitaya seedlings exhibited diverse characteristics (Figure 8B). Using root expression levels as a reference (set to 1), we found that some genes showed obvious tissue-specific expression. Except for *HuSRS9*, which showed extremely high transcriptional accumulation in stem segments, most genes were highly expressed in cotyledons. The relative expression levels of *HuSRS1*, *HuSRS5*, *HuSRS7*, and HuSRS8 in cotyledons were all over 50 times higher than those in roots (set as the baseline at 1.0). *HuSRS9* exhibited tissue-specific expression, with levels remaining close to the baseline in roots (~1.02) but showing a substantial increase of approximately 40.50-fold in cotyledons. Its high expression in stem segments suggests a potential association with basal metabolism of young stem elongation.

## 4. Discussion

The *SRS* gene family, a group of plant-specific transcription factors, plays crucial roles in plant growth, development, and responses to biotic and abiotic stresses. While extensively studied in various plant species, the *SRS* family remains uncharacterized in pitaya (*Hylocereus undatus*). This study provides a comprehensive bioinformatic and expression analysis of the *HuSRS* gene family [35,36].

Through genome-wide scanning and bioinformatics analysis, nine members of the *HuSRS* gene family (*HuSRS1* to *HuSRS9*) were identified. These genes are widely distributed in the pitaya genome, with a quantity fewer than the 16 in the model plant Arabidopsis [37], but more than the 6 in monocotyledonous maize [13], indicating that the *SRS* gene family has undergone expansion or contraction in different plant species. Most HuSRS proteins are predicted to be unstable and hydrophilic based on in silico analysis. Subcellular localization prediction shows that the nine family members are mainly localized in the nucleus, with HuSRS3 and HuSRS4 in chloroplasts and HuSRS6 in the cell membrane, suggesting diverse functional localizations of HuSRS family members within cells. Collinearity analysis revealed that 8 *HuSRS* genes formed 13 pairs of homologous gene pairs through segmental duplication, with Ka/Ks < 1, indicating that purifying selection has maintained gene functional stability. This finding is consistent with studies in poplar, demonstrating that purifying selection has been applied to the *SRS* gene family during evolution [38].

Phylogenetic tree analysis indicated that the *HuSRS* gene family can be divided into three subclasses, with genes within the same subclass exhibiting similar structures but certain variations, which may be associated with the functional diversity of *SRS* genes and their evolutionary changes. Exon-intron structure analysis of *HuSRS* genes showed consistency with reported *SRS* genes in rice [39] and *Melilotus albus* [40], which contain at least one exon. Notably, HuSRS2, HuSRS4, and HuSRS5 lacked UTRs at both ends. Conserved motif analysis revealed that Motif 1, Motif 2, and Motif 3 were shared among all HuSRS family members. Specifically, Motif 1 contained a RING-type zinc finger domain, and Motif 2 harbored an IXGH domain, both of which were conserved in all HuSRS proteins—consistent with reports that all HuSRS proteins in Arabidopsis possess both ring zinc finger and IXGH domains [41]. However, amino acid sequence alignment and conserved domain analysis in maize showed that only 10 of the 13 maize SRS family proteins contained both RING and IXGH domains [13].

Phylogenetic tree construction by comparing with Arabidopsis and rice *SRS* gene families revealed closer evolutionary relationships between *HuSRS* genes and rice *SRS* genes, indicating high homology between the two. Based on these findings, we speculate that *HuSRS* family members may share similar biological functions with rice *SRS* genes, providing important clues and theoretical basis for further deciphering the regulatory mechanisms of *HuSRS* genes in pitaya growth and development.

Secondary and tertiary structure predictions revealed that HuSRS proteins are primarily composed of random coils, α-helices, and extended strands. Spatial structures of HuSRS1-HuSRS2 and HuSRS7-HuSRS8 pairs showed high similarity, whereas HuSRS4 and HuSRS5 exhibited shorter and simpler architectures. Genes with similar spatial structures all belonged to the same subclass, indicating that sequence conservation determines structural similarity to some extent—a finding consistent with studies in *Melilotus albus* [40]. Such structural similarity is likely closely associated with functional similarity, suggesting that these proteins may have experienced similar selective pressures during evolution, thus retaining analogous structural features to execute conserved biological functions. Based on HuSRS protein interaction data, a complex PPI network was identified, where YUC4 and F6N18.11 interacted with multiple family members—consistent with previous findings in *Sesamum indicum* [42]. YUC4, a key rate-limiting enzyme catalyzing plant auxin biosynthesis, exhibits multiple regulatory functions in the developmental processes of *Arabidopsis thaliana* [43]. HuSRS8 protein interacts with MED19B, EIF4B3, rpl5, and Q1PDX4_ARATH. These proteins are involved in key processes of plant growth and development in Arabidopsis, such as gene transcriptional regulation, protein synthesis, and material metabolism. These interaction relationships indicate that HuSRS8 protein may play a hub role in transcriptional regulation, protein synthesis, and maintenance of metabolic homeostasis. HuSRS2 protein interacts with AHL18 and Q8LFJ2_ARATH. AHL18 (an AP2/ERF family transcription factor) has been confirmed to be involved in the regulation of plant cell proliferation and plays a key regulatory role in cell division and organ morphogenesis [44]. Q8LFJ2_ARATH regulates cell elongation and differentiation by participating in hormone signal transduction pathways. Based on these findings, it is plausible to speculate that the HuSRS2 protein may be involved in processes such as cell division regulation, organ development, and hormone response. HuSRS7 protein interacts with Q9LUI0_ARATH, potentially involved in regulating cellular material transport, metabolic balance, and organ development. HuSRS1 protein interacts with NGA3 (NAC-Like, Activated by AP3/PI) [45], a member of the NAC transcription factor family in Arabidopsis that participates in regulating leaf senescence, light signal transduction, and plant stress resistance—consistent with findings in *quinoa* (*Chenopodium quinoa*) [46]. These interactions indicate that HuSRS proteins play important roles in various aspects of growth and development, contributing to a deeper understanding of the molecular basis of pitaya biology and providing directions for improving pitaya growth and stress resistance.

Based on transcriptome data analysis, *HuSRS* genes exhibited differential expression patterns during the development of pitaya flowers, flesh, and peel. During flower development, eight genes except *HuSRS5* were expressed, with *HuSRS6* showing significantly higher expression at the flower bud differentiation stage than other stages, indicating its potential key regulatory role in flower bud differentiation. The results were consistent with previous studies in *Arabidopsis thaliana*, where *SRS* genes were highly expressed in flowers and roots [47]. During flesh development, only *HuSRS1*, *HuSRS4*, *HuSRS6*, and *HuSRS7* were expressed. The expression level of *HuSRS4* significantly increased at 29 days of flesh development, suggesting its potential important function in specific stages of flesh development. During peel development, eight genes except *HuSRS4* were expressed, with the expression level peaking at 65 days of peel development. Among them, *HuSRS6* maintained a relatively high expression level throughout the peel development process, possibly playing an important role in peel maturation.

*Cis*-acting element analysis of promoters revealed that *HuSRS* gene promoters predominantly contain light-responsive and hormone-responsive elements. Combining with expression patterns, it is speculated that *HuSRS* genes may participate in organ development by responding to photoperiod and hormone signals (such as abscisic acid and jasmonic acid). Additionally, the promoter regions of the *HuSRS* gene family also contain *cis*-acting elements related to meristem expression, anaerobic induction, and zein metabolism regulation, which is consistent with findings in cassava [48].These findings indicate that the *HuSRS* family exerts multifaceted regulatory functions in pitaya growth and development.

Using real-time quantitative PCR, we analyzed *HuSRS* gene expression in cotyledons, stem segments, roots, and callus of pitaya seedlings, while measuring physiological indices of callus tissues. Results showed that the WG group exhibited significantly higher soluble protein content and lower electrical conductivity than the CK group, indicating active cellular metabolism and stable membrane systems in WG callus. All *HuSRS* genes showed higher expression in WG than CK callus, with *HuSRS4*, *HuSRS5*, and *HuSRS7* having relative expression levels >100. These genes may accelerate callus formation by potentially promoting cell dedifferentiation and division. Similarly, 23 *SRS* genes were preferentially expressed in rapeseed (*Brassica napus*) callus, supporting conserved roles in plant somatic tissue development [49]. The ABRE elements in the *HuSRS5* promoter are associated with its high expression in callus, suggesting that abscisic acid signaling may be involved in regulating its role in cell dedifferentiation [50]. *HuSRS* genes may accelerate callus formation by promoting cell dedifferentiation and division. In vegetative organs of seedlings, *HuSRS* genes exhibit tissue-specific expression: *HuSRS9* shows extremely high relative expression in stem segments (>800), potentially associated with stem elongation metabolism; *HuSRS1*, *HuSRS5*, *HuSRS7*, and *HuSRS8* are highly expressed in cotyledons (>50). The high expression of *HuSRS8* in cotyledons may be related to its encoded protein mediating light-responsive gene expression via interaction with *MED19B*. Compared with roots and stem segments, most *HuSRS* genes show significant expression differences in cotyledons, indicating that *HuSRS* genes may have unique regulatory networks during cotyledon development to adapt to the functional requirements of cotyledons as primary organs for photosynthesis and material storage.

It should be noted that the callus tissue used in this study is an in vitro-induced aggregate of undifferentiated cells, whose gene expression profiles fundamentally differ from those of differentiated tissues in intact plants. Callus lacks true tissue structure and organ characteristics, and its growth is highly dependent on exogenous hormones and culture conditions. Therefore, experimental results derived from this system have significant limitations when directly extrapolated to the overall physiological functions of the plant. The primary objective of using callus in this study was to leverage its characteristics of active cell division and rapid gene expression responses to preliminarily screen *HuSRS* genes that may play key roles in cell proliferation and differentiation.

Despite the rigorous experimental design of this study, a limitation should be considered when interpreting the qPCR results. In this research, relative gene expression was normalized using a single reference gene, *HuActin*, which was selected based on its widespread use in previous studies on pitaya [51,52] and validated for amplification specificity under our experimental conditions via melting curve analysis.

## 5. Conclusions

The *HuSRS* gene family exhibits spatiotemporal-specific expression patterns and diversified regulatory functions during pitaya growth and development. Its members play critical roles in tissue differentiation, organ development, and environmental adaptation by participating in pathways such as cellular metabolism, hormone response, and stress response. This study not only provides a theoretical basis for deciphering the functions of *HuSRS* genes but also lays a foundation for molecular breeding, efficient callus induction, and stress-resistant genetic improvement in pitaya. Follow-up research can deeply validate the biological functions of each member through technical approaches like gene editing and overexpression, explore their application potential in pitaya quality enhancement and stress adaptation, and promote the innovative development of molecular breeding technologies in the pitaya industry.

## Figures and Tables

**Table 1:** Predicted subcellular localization of HuSRS proteins.

Member Name	ID	Number of Amino Acids	Molecular Weight/Da	pI	Hydrophobicity Coefficient	Subcellular Localization
HuSRS1	HU01G00264	235	24883.69	9.13	−0.528	Nucleus
HuSRS2	HU03G00149	255	27036.96	8.98	−0.509	Nucleus
HuSRS3	HU05G02071	289	31034.41	6.14	−0.62	Chloroplast
HuSRS4	HU06G00092	326	34471.12	8.06	−0.637	Chloroplast
HuSRS5	HU07G00744	357	38872.62	6.92	−0.807	Nucleus
HuSRS6	HU08G00953	365	36488.21	8.68	−0.336	Cell membrane
HuSRS7	HU08G01189	351	36500.44	9.04	−0.565	Nucleus
HuSRS8	HU08G01241	351	36540.51	9.04	−0.569	Nucleus
HuSRS9	HU11G01269	356	38240.04	7.18	−0.802	Nucleus

**Table 2:** Secondary structure prediction of HuSRS proteins.

Protein	Alpha Helix	Extended Strand	Random Coil
HuSRS1	8.51%	8.09%	83.4%
HuSRS2	10.20%	8.24%	81.57%
HuSRS3	8.65%	8.30%	83.04%
HuSRS4	11.35%	10.74%	77.91%
HuSRS5	6.72%	9.24%	84.03%
HuSRS6	6.85%	7.12%	86.03%
HuSRS7	8.83%	6.27%	84.90%
HuSRS8	10.54%	9.97%	79.49%
HuSRS9	7.02%	6.18%	86.80%

## Data Availability

The transcriptome sequencing data of the *HuSRS* gene family in pitaya from this study are available in the Pitaya Genome and Multiomics Database (http://pitayagenomic.com/ (accessed on 17 March 2025)). All datasets generated and analyzed during this study, including gene expression profiles and functional annotation data, are included within this published article and Supplementary Materials.

## Supplementary Materials

The following supporting information can be downloaded at: https://www.mdpi.com/article/10.3390/plants14203139/s1, Table S1 Sequences of SRS member proteins from six species; Table S2 Primers used in this study.
